# A Detachable Integrated 183 GHz Terahertz Low-Noise Amplifying and Mixing Frontend

**DOI:** 10.3390/mi17050562

**Published:** 2026-04-30

**Authors:** Qiyuan Zheng, Jin Meng, Li Wang, Zhaoyue Wang

**Affiliations:** 1 The CAS Key Laboratory of Microwave Remote Sensing, National Space Science Center, Chinese Academy of Sciences, Beijing 100190, China; zhengqiyuan23@mails.ucas.ac.cn (Q.Z.); wangli1@mirslab.cn (L.W.); wangzhaoyue23@mails.ucas.ac.cn (Z.W.); 2 University of Chinese Academy of Sciences, Beijing 100049, China

**Keywords:** terahertz, detachable integration, low-noise amplifier, second-order subharmonic mixer

## Abstract

Conventional terahertz (THz) radio frequency (RF) frontends struggle to simultaneously balance the high performance and miniaturization of monolithic integrated designs with the excellent testability of discrete modular architectures. This paper presents a detachable 183 GHz terahertz RF frontend and completes the module design and system integration of a low-noise amplifier (LNA) and a second-order subharmonic mixer. Through optimization of the waveguide-to-microstrip transition, parasitic compensation for pad bonding, and the structural design of the chip shielding cavity, combined with a high-precision alignment scheme using positioning pins and screws, the integrated module achieves detachability, testability, and ease of maintenance. Measurement results show that across the 160–200 GHz frequency band, the amplifier achieves an average gain of 16.51 dB; the mixer exhibits a minimum conversion loss of 8.62 dB; and the full-link noise figure of the system reaches 6.68 dB. The proposed scheme effectively addresses the engineering challenges of conventional integrated architectures and provides a practical implementation pathway for terahertz communication and remote sensing detection frontends.

## 1. Introduction

The terahertz (THz) band lies between the microwave and infrared regions. Owing to its merits of large bandwidth, high resolution, low photon energy and material fingerprint spectral characteristics, it exhibits significant application value in 6G communication, radar detection, atmospheric remote sensing, precision metrology and other fields [1,2,3,4,5,6,7,8,9]. As the core component of a terahertz receiving system, the radio frequency (RF) frontend undertakes key functions such as signal amplification and frequency conversion. Its integration level, high-frequency performance and maintainability directly determine the engineering implementation capability of the entire system.

In existing architectures, terahertz RF frontends are mainly realized by two schemes: modular independent packaging and multi-chip monolithic packaging. The modular independent packaging scheme features high unit independence and facilitates individual testing and replacement, but suffers from large system volume. The multi-stage assembly tends to increase inter-stage interconnection loss, making it difficult to meet the requirements of high performance and miniaturization. The multi-chip monolithic packaging scheme can effectively reduce the volume and transmission loss to achieve superior high-frequency performance. However, the modules are non-detachable, leading to difficulties in fault location and high maintenance costs, which cannot adapt to the demands of flexible debugging and rapid maintenance in engineering applications. Both traditional architectures struggle to simultaneously satisfy the comprehensive requirements of high performance, testability and easy maintenance.

As a critical atmospheric window frequency band, 183 GHz is characterized by low transmission loss and is suitable for long-range radiation detection and high-speed communication, posing stringent requirements on the sensitivity, bandwidth and stability of the receiving frontend. At present, domestic and international research mostly focuses on chip performance improvement and monolithic integrated design, while studies on detachable, maintainable and low-loss terahertz RF frontends are still insufficient.

At present, domestic and international institutions have carried out extensive research on RF frontend integration technologies. The technical system has evolved along the trajectory from foundational maturity in the microwave and millimeter-wave bands to high-frequency adaptation and innovation in the terahertz band. The research progress and technical limitations of the two bands are summarized as follows:

The microwave and millimeter-wave bands represent the mature application spectrum for RF integration technologies. Its core development trajectory has evolved from early 2D planar integration technology, gradually to 2.5D heterogeneous packaging integration and 3D high-density three-dimensional integration. The core objective is to balance integration level, transmission performance and engineering cost within the framework of mature processes, while also providing a reference for the development of integration technologies for the high-frequency terahertz band.

At present, representative research in this band focuses on the design of high-density integrated architectures. Wan Tao et al. [10]. adopted low-temperature co-fired ceramic (LTCC) and ball grid array (BGA) packaging technologies, combined with chip integration and optimized quasi-coaxial vertical interconnection design, to develop a miniaturized 3D Ku-band transceiver module. The module achieves an output power higher than 24.5 dB, a receive gain greater than 25 dB, and a receive noise figure lower than 3.5 dB within the 11–17 GHz band. This research is a typical application of 2.5D packaging integration technology, which solves the problems of large volume and high inter-stage cascade loss of traditional planar integration, and achieves a balance between integration level and performance. However, such solutions rely on vertical wiring in multi-layer substrates. When the operating frequency is increased to the terahertz band, the parasitic effects and transmission loss of vertical interconnections will increase exponentially, making them unable to adapt to high-frequency requirements.

Zhao Yu et al. [11]. adopted silicon-based MEMS 3D heterogeneous integration, through-silicon via (TSV) and ultrasonic thermocompression gold bump interconnection technologies to develop an 8-beam 32-channel tile-type phased array transmit frontend. The frontend achieves a single-channel transmit gain higher than 18 dB, an input/output port standing wave ratio (SWR) less than 2.0 in the 19–21 GHz band, and integrates 6-bit digital phase shifting and 5-bit digital attenuation functions. This research represents a leading level of high-density integration in the millimeter-wave band, achieving micron-level assembly accuracy and ultra-high integration density through MEMS micromachining. However, this solution has a high process threshold and expensive fabrication cost, and adopts an integrated monolithic architecture, which cannot realize independent testing and maintenance of functional modules. Meanwhile, the dielectric loss of the silicon-based substrate increases significantly in the terahertz band, making it difficult to be directly migrated to high-frequency terahertz applications.

Overall, a mature multi-dimensional integration technology system has been formed in the microwave and millimeter-wave bands, realizing multi-dimensional optimization of integration level, performance and cost. However, when existing solutions are migrated to the high-frequency terahertz band, they will face severe degradation of transmission loss and parasitic effects, making it difficult for relevant technologies to be directly applied to the terahertz band.

In the terahertz band, aiming at the high-frequency loss bottleneck encountered when migrating microwave and millimeter-wave integration technologies to the terahertz band, domestic and international research mainly focuses on the monolithic integration of modules at the circuit level, while the research and application of chip-level high-density integration are still relatively insufficient. Its core objective is to solve the problems of high transmission loss, excessive size and high engineering cost of traditional discrete architectures in the high-frequency band.

Representative studies are as follows: Niu Zhongqian et al. [12]. proposed a terahertz 3D stacked vertical interconnection technology based on coaxial probes, realizing the miniaturized integration of terahertz RF frontends. Simulation results show that the mixer exhibits a conversion loss of less than 9 dB in 195–230 GHz, and the frequency multiplier delivers a broadband output power higher than 4 mW in 104–116 GHz with a 90 mW input. This research verifies the high-performance potential of 3D vertically stacked architectures in the terahertz band.

B. Thomas et al. [13] integrated a 520–600 GHz sub-harmonic mixer and a 260–300 GHz tripler into a single cavity based on GaAs monolithic microwave integrated circuit (MMIC) membrane planar Schottky diode technology, using both traditional metal milling and silicon micromachining processes to achieve high-density integration of the terahertz RF frontend. The metal-machined module achieves a mixer noise temperature of 2360 K and a conversion loss of 7.7 dB at 520 GHz, while the silicon-micromachined module obtains a noise temperature of 4860 K and a conversion loss of 12.16 dB at 540 GHz. This research is a typical representative of monolithic integration in the high-frequency terahertz band and achieves excellent high-frequency performance.

The advantages and limitations of several RF frontend integration technologies are compared in Table 1. Most existing studies are currently oriented towards monolithic fixed integration architectures. Such structures have a fixed integration level, with all chips permanently packaged in the same cavity. The failure or required upgrade of any functional unit may lead to the scrapping of the entire module, resulting in poor maintainability and flexibility. Therefore, conducting research on the key technologies of detachable RF integrated frontends and breaking through the inherent limitations of traditional integration architectures is of great significance for promoting the development of terahertz systems towards practicalization and high reliability.

This paper proposes a 183 GHz detachable terahertz RF frontend scheme, which takes a low-noise amplifier (LNA) and a second-order subharmonic mixer as the core modules, and carries out module design and system integration. The high-frequency performance of the core modules is improved through the optimization of the waveguide-to-microstrip probe transition, bonding parasitic compensation, shielding cavity resonance suppression, and high-low impedance low-pass filter network. A high-precision docking structure combining positioning pins and screws, together with standard waveguide transition interfaces, is adopted to realize the detachability, testability and ease of maintenance of the modules. Finally, the fabrication, assembly and full-link testing are completed, which provides a practical and feasible implementation scheme for high-performance, maintainable terahertz receiving frontends.

## 2. Design of Circuits and Structures

This section focuses on the detachable 183 GHz THz RF frontend, conducting core circuit optimization of the LNA and second-order subharmonic mixer, respectively, along with the design of the detachable integrated structure.

### 2.1. Low-Noise Amplifier Design

The amplifier adopts the TCC1967N D-band broadband amplifier chip, which is in the form of an unpackaged bare die. It covers an operating frequency band of 120–200 GHz, with a typical small-signal gain of 20 dB and a noise figure of 3.5 dB, which meets the low-noise amplification requirements of the frontend. In view of the bare die form, operating frequency band and port characteristics of the chip, the core work of this design mainly focuses on the following three aspects: first, the design of the shielding cavity and electromagnetic isolation structure, which addresses the performance degradation and even self-oscillation of the chip caused by cavity resonance and electromagnetic leakage in the terahertz band; second, the design of the waveguide-to-microstrip probe transition structure, to realize low-loss signal transmission between the waveguide and the microstrip; third, the design of the bonding wire parasitic parameter compensation network, to cancel the parasitic inductance introduced by conventional gold wire bonding in the terahertz band and avoid port impedance mismatch.

**Figure 1:**
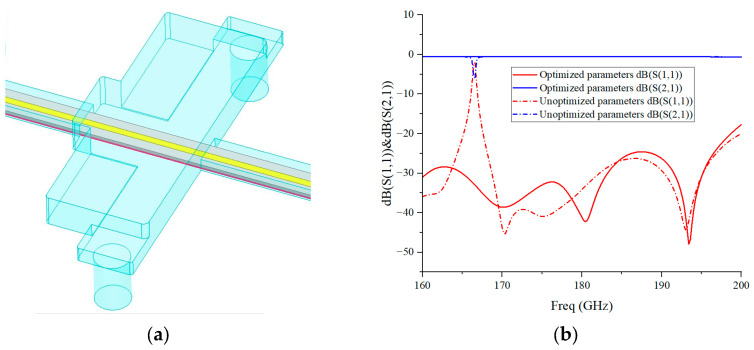
(**a**) The 3D model of the optimized shielding cavity structure; (**b**) simulated S-parameters.

To ensure the signal integrity and electromagnetic compatibility of the LNA in the terahertz band, the LNA adopts a fully enclosed metallic shielding cavity structure. The chip, matching network and transmission circuit are all enclosed within the metallic cavity to suppress signal radiation leakage and block external electromagnetic interference. A feed-through capacitor biasing structure is utilized to completely isolate the DC power supply from the RF cavity, thereby interrupting parasitic coupling paths. A simulation model of the passive shielding cavity is established in the Ansys HFSS 2022 R1 simulation software, and the cavity structure is optimized for passive transmission performance. The optimized model of the shielding cavity structure and the simulated S-parameters are shown in Figure 1. Simulations demonstrate stable transmission performance within the 160–200 GHz band, with a return loss better than 25 dB, an insertion loss lower than 0.5 dB, and no obvious resonant interference, which provides a favorable electromagnetic operating environment for the chip.

**Figure 2:**
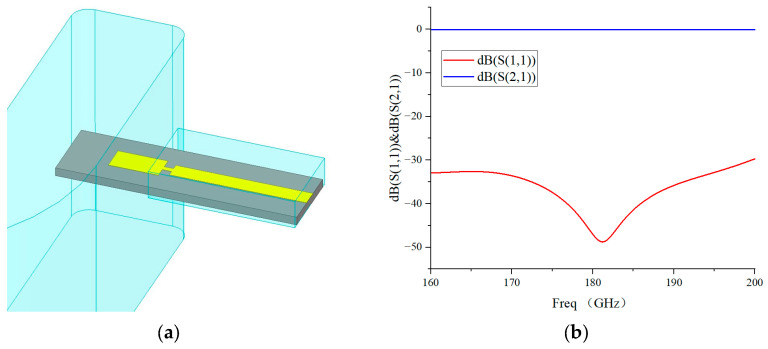
(**a**) The 3D model of the E-plane probe transition structure; (**b**) simulated S-parameters of the waveguide-to-microstrip transition.

In this design, the widely adopted E-plane probe transition is utilized [14,15] to achieve efficient mode conversion between the WR-5 waveguide TE_10_ mode and the microstrip quasi-TEM mode. The probe is placed at λ_g/4 away from the short-circuit plane to achieve maximum coupling strength, where λ_g represents the guided wavelength at 183 GHz, approximately 1.1 mm, ensuring maximized coupling efficiency. Broadband low-loss matching is realized by optimizing the probe length, matching line width and high-impedance line structure. Its 3D model and simulated S-parameters are illustrated in Figure 2. Simulation results show that the return loss of the transition structure is better than 30 dB and the insertion loss is less than 0.12 dB within 160–200 GHz.

**Figure 3:**
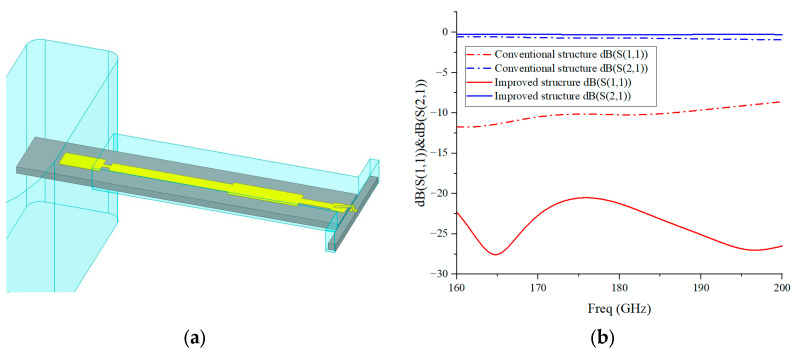
(**a**) The 3D model of the bonding structure with the improved compensation network; (**b**) simulated S-parameters of the bonding structure with the compensation network.

Gold wire bonding is used for the connection between the microstrip line and the chip pads. However, gold wire bonding exhibits significant series inductance in the terahertz band, degrading port matching. The return loss of conventional direct bonding is only 8–9 dB. In this work, a high-low impedance line compensation network is introduced to cancel the bonding parasitic reactance. Its 3D model and simulated S-parameters are presented in Figure 3.

### 2.2. Design of the Second-Order Subharmonic Mixer

The second-order subharmonic mixer uses an antiparallel Schottky diode pair (AP1/G2/0P95) as the nonlinear device and realizes frequency mixing through the rectification and conductance modulation effects of the diodes [16]. Under the joint excitation of the radio frequency (RF) small signal and the local oscillator (LO) large signal, the total conductance of the diode pair only contains the direct current (DC) and even-order harmonic components, so that the intermediate frequency (IF) signal satisfying Equation (1) can be extracted.

(1) $$f_{\mathrm{IF}}=\left|f_{\mathrm{RF}}-2f_{\mathrm{LO}}\right|$$

**Figure 4:**
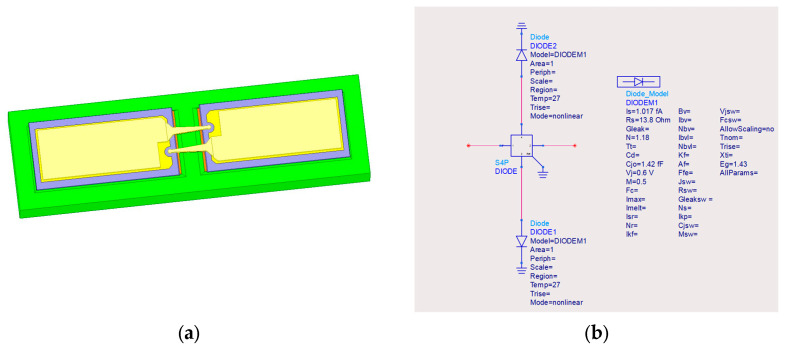
(**a**) The 3D model of the diode pair in HFSS; (**b**) equivalent circuit model of the diode pair in ADS.

To accurately characterize the parasitic effects of the diode in the terahertz band, a hybrid modeling method combining full-wave electromagnetic modeling in HFSS and nonlinear circuit simulation in ADS is adopted. First, a full-wave electromagnetic model of the diode is established to extract package parasitic parameters. These parameters are then imported into the circuit simulation software, and parameter calibration is performed, combined with measured I–V data to obtain an accurate nonlinear simulation model. The final obtained model of the diode pair is shown in Figure 4.

Key parameters of the calibrated diode are as follows: series resistance $R_{s}=13.8\;\Omega$, ideality factor η=1.18, zero-bias junction capacitance $C_{j0}=1.42\times 10^{-15}F$. The cut-off frequency of the diode is calculated by Equation (2) as: $f_{c}$ = 8.62 THz, which is much higher than the operating frequency band, thus ensuring excellent high-frequency nonlinear performance.

(2) $$f_{c}=\frac{1}{2\pi R_{s}C_{j0}}$$

**Figure 5:**
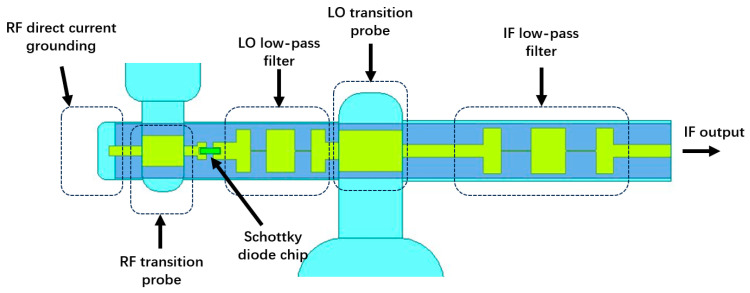
Topology of the mixer.

The mixer adopts a topology with dual waveguide inputs (RF/LO), a suspended microstrip circuit, and an SMA output for the IF signal, as shown in Figure 5.

**Figure 6:**
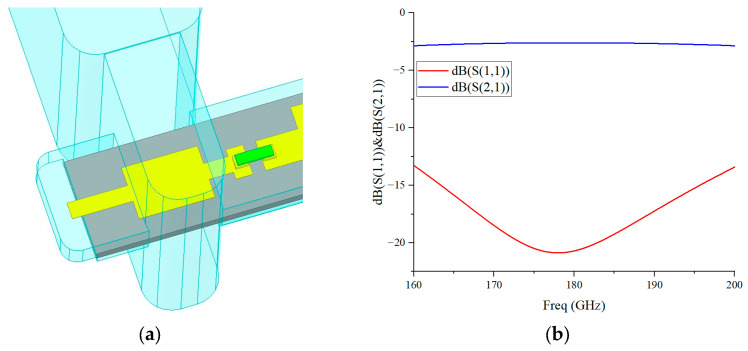
(**a**) The 3D model of the waveguide-to-microstrip probe transition; (**b**) simulated S-parameters.

The mixer design also uses an E-plane probe transition to achieve high-efficiency coupling from the waveguide TE_10_ mode to the suspended microstrip. Broadband impedance matching is realized by optimizing the probe length, matching line width, and short-circuit terminal position. The 3D model and simulation results of the transition structure are shown in Figure 6.

Simulation results show that the transition structure exhibits a return loss better than 13 dB and an insertion loss lower than 2 dB within 160–200 GHz, meeting the requirements of broadband low-loss transmission.

**Figure 7:**
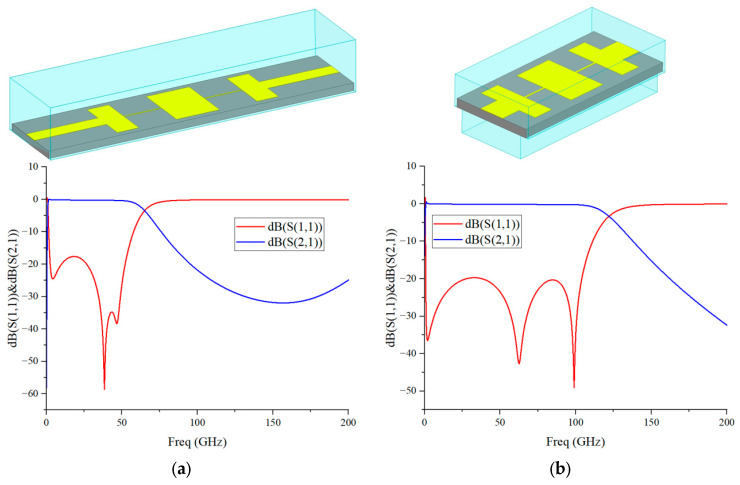
(**a**) The 3D structure and Simulated S-parameters of the IF low-pass filter; (**b**) 3D structure and Simulated S-parameters of the LO low-pass filter.

To achieve high isolation among the three ports, an IF low-pass filter and a LO low-pass filter are designed, respectively. Both filters adopt a high-low impedance line structure to realize steep out-of-band rejection and compact size, and their models and simulated results are shown in Figure 7.

The IF LPF provides a rejection higher than 13 dB in the stopband of 80–200 GHz, an insertion loss lower than 0.4 dB, and a return loss better than 17 dB in 0–50 GHz. The LO LPF achieves a rejection higher than 20 dB in 160–200 GHz, an insertion loss lower than 0.2 dB, and a return loss better than 20 dB in 0–100 GHz. The two-stage filters work synergistically to effectively suppress RF and LO leakage as well as higher-order harmonic interference, significantly improving port isolation.

**Figure 8:**
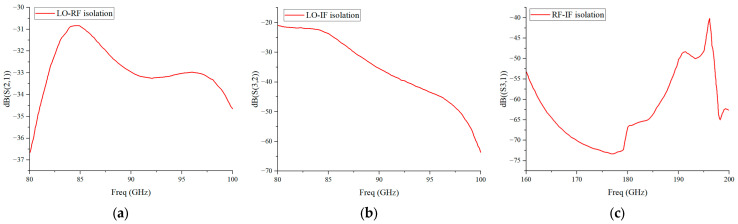
(**a**) Simulation results of LO-RF port isolation; (**b**) Simulation results of LO-IF port isolation; (**c**) Simulation results of RF-IF port isolation.

To verify the design effectiveness of the low-pass filters, the isolation simulation of the RF, LO and IF ports of the mixer is completed based on ADS, with the simulation results shown in Figure 8. It can be seen that the LO-RF isolation(S(2,1)) is better than 30 dB, the LO-IF isolation(S(3,2)) is better than 20 dB, and the RF-IF isolation(S(3,1)) is better than 40 dB. The simulation results validate the design effectiveness of the LO and IF low-pass filters, which can ensure the low-crosstalk operation between the ports of the mixer.

### 2.3. Design of the Detachable Integrated Structure

**Figure 9:**
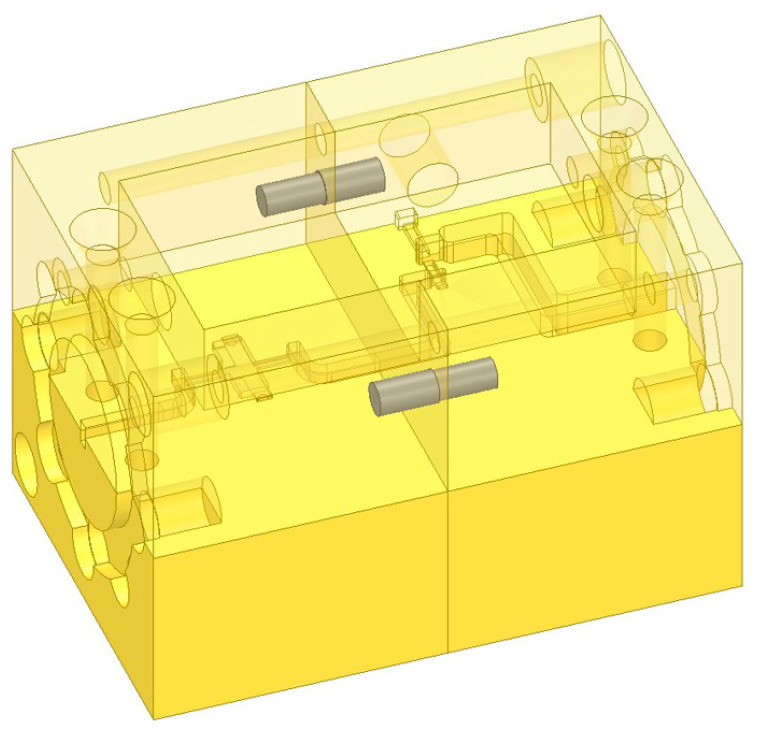
Schematic of the integrated structure.

The integrated system adopts a split layout along the radio-frequency signal propagation direction, and the structural schematic is shown in Figure 9. The low-noise amplifier and mixer modules are arranged in sequence. The radio-frequency signal is fed into the LNA through a WR-5 waveguide and then connected to the mixer via an embedded short waveguide inside the module. The local oscillator and intermediate-frequency signals are input and output through the WR-10 waveguide port on the side of the mixer and the SMA interface at the end, respectively.

**Figure 10:**
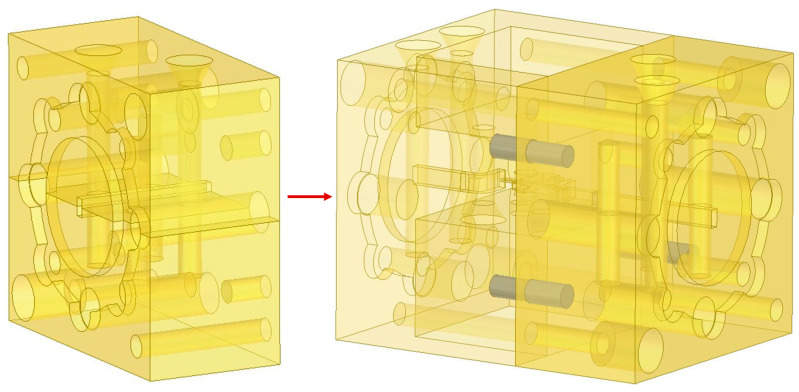
Schematic of the adapter structure.

A combined structure consisting of high-precision positioning pins and M1.6 miniature fastening screws is adopted between modules to achieve bidirectional precise alignment in both radial and axial directions, as well as robust connection of the detachable structure. Meanwhile, matched test adapter interfaces are designed for the module connection cross-section, as shown in Figure 10, which can be directly connected to standard flange-mounted test equipment. The adapters are fastened by positioning pins and screws and support back-to-back calibration, satisfying the requirements of both independent module testing and system integration testing.

## 3. Module Assembly and Measurement

The cavity of the integrated frontend is fabricated from gold-plated brass. The circuit substrate adopts 50 μm-thick quartz with a relative dielectric constant of 3.78. The assembly follows a sequential procedure of individual module assembly first, followed by system integration: die bonding, passive circuit assembly, and gold wire bonding are completed separately for the low-noise amplifier and the second-order subharmonic mixer. The two-stage modules are then precisely aligned radially and axially by means of positioning pins and firmly assembled using four M1.6 screws (Tiancheng Hardware Co., Ltd., Dongguan, China). The machining tolerance of the positioning pins is ±5–10 μm, which ensures docking accuracy and mechanical reliability. The adapter structure is fabricated using the same process as the cavity, with positioning pins and threaded holes designed at the mating interface with the cavity, to ensure the connection method is consistent with the integrated connection of the cavity modules.

**Figure 11:**
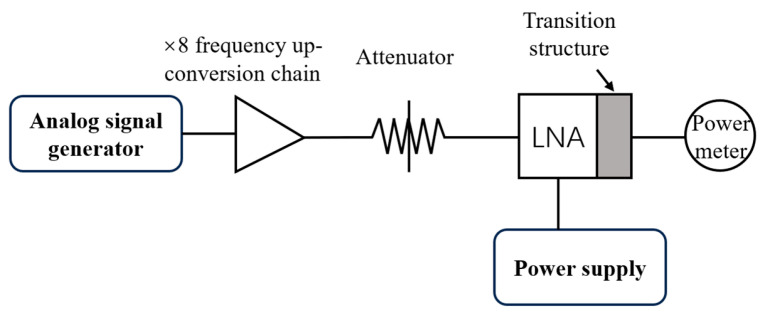
LNA gain test block diagram.

**Figure 12:**
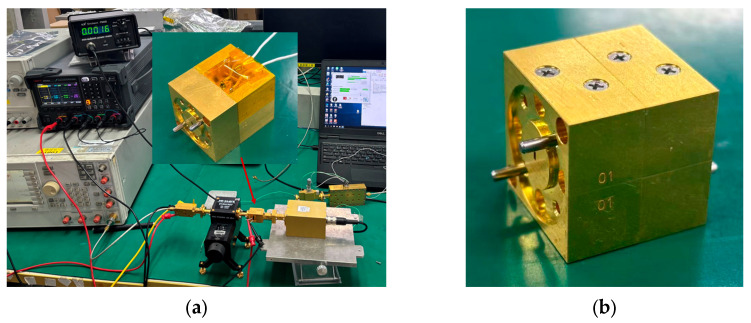
(**a**) Photograph of the LNA module and the gain measurement setup; (**b**) measured gain of the LNA module.

The schematic diagram of the LNA gain test platform is shown in Figure 11. A signal source combined with an eighth-frequency multiplier chain is used to provide the excitation signal in the 160–200 GHz band; the input power is regulated by a variable attenuator, and the output power is measured by a power meter. The photograph of the assembled test platform is shown in Figure 12a. Notably, the output port of the LNA module is interfaced with the power meter through the adapter structure. To ensure the experimental rigor and the feasibility of the integrated structure design, the power loss of the adapter structure must also be tested. The test procedure is consistent with the aforementioned LNA test procedure, only replacing the LNA module under test with the back-to-back assembled adapter structure shown in Figure 12b.

**Figure 13:**
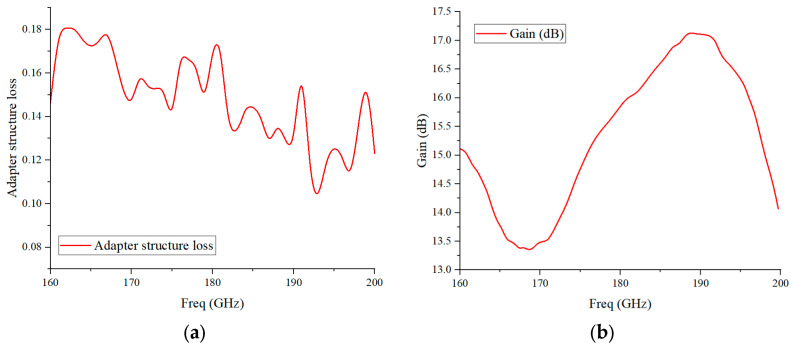
(**a**) Test results of the adapter structure loss; (**b**) test results of the LNA module gain.

The test results of the adapter structure loss and the corrected gain measurement results of the LNA module are shown in Figure 13. It can be seen that the back-to-back adapter structure assembled with positioning pins and screws has an average loss of 0.16 dB, which has a negligible impact on the module performance. Within the 160–200 GHz operating band, the LNA module achieves an average gain of 16.51 dB and a maximum gain of 17.1 dB.

The in-band fluctuation observed in the measured gain curve mainly originates from various non-ideal factors in the engineering implementation and testing process of the module. In this design, gold wire bonding is adopted to realize the interconnection between the chip and the microstrip line. Although a bonding parasitic compensation network has been designed to optimize the nominal matching characteristics, there are micron-scale deviations in the length and arch height of the bonding wires during actual assembly. These deviations are comparable to the operating wavelength and thus non-negligible, which will lead to frequency-dependent variation in the bonding parasitic inductance and cause impedance mismatch at different frequency points in the band. Meanwhile, deviations between the fabricated shielding cavity (including dimensional tolerance, metal surface roughness, and assembly sealing performance) and the ideal simulation model may induce residual high-order mode resonance in the operating band. In addition, the assembly deviation of the waveguide-to-microstrip transition structure will also result in differences in in-band transmission loss. The above multiple factors lead to the deviation between the measured results and the on-wafer test results of the bare chip.

**Figure 14:**
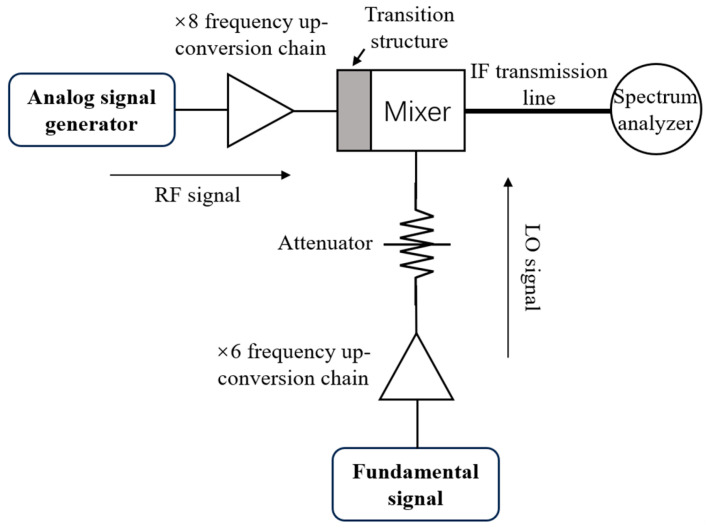
Mixer test block diagram.

**Figure 15:**
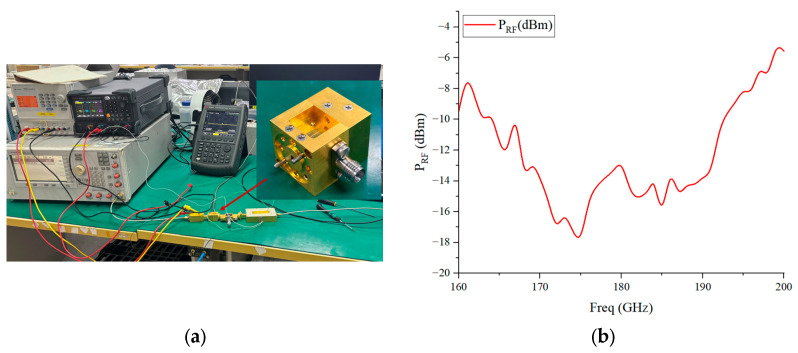
(**a**) Photograph of the mixer module test setup; (**b**) measured input signal power at each test frequency point.

The schematic diagram of the performance test platform for the mixer module is shown in Figure 14. The RF signal in the 160–200 GHz band is provided by an RF signal source combined with an eighth-frequency multiplier chain, while the 91 GHz LO signal is supplied by a local oscillator source combined with a sixth-frequency multiplier chain. The signal power is adjusted to 6 mW via an attenuator to provide the required LO drive signal. The photograph of the assembled test platform is shown in Figure 15a.

During the test, the spectrum analyzer is first connected to the output port of the eighth-frequency multiplier chain at the RF end to calibrate the input signal power at each test frequency point. The measured input signal power is shown in Figure 15b. Subsequently, the mixer module under test is connected to the link, and the IF output port of the mixer is connected to the spectrum analyzer via a transmission line to read the IF output power. To ensure experimental rigor, the effects of the loss of the IF output transmission line and the adapter structure on the test results are also taken into account. The specific loss data of the transmission line is measured by connecting one end of the transmission line to a signal generator and the other end to the spectrum analyzer, then reading the difference between the input and output power. The influence of the adapter structure loss on the test is reflected at the RF signal input end: the actual input power at the RF port is obtained by subtracting the loss power of the adapter structure from the calibrated input signal power at each test frequency point.

**Figure 16:**
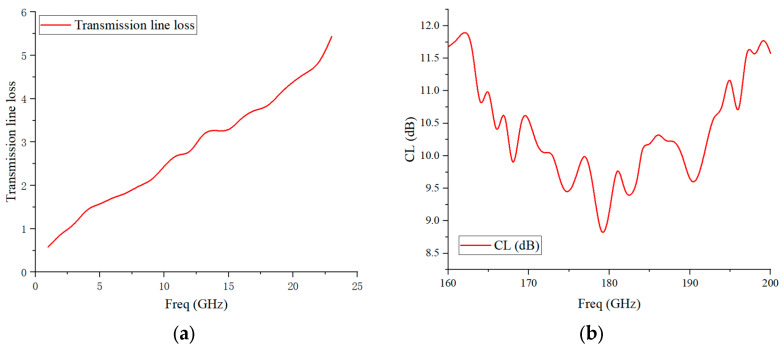
(**a**) Test results of transmission line loss; (**b**) corrected test results of mixer conversion loss.

The test results of the IF transmission line loss and the corrected mixer measurement results are shown in Figure 16. The measurement results show that when the LO frequency is fixed at 91 GHz, the mixer achieves an average conversion loss of 10.37 dB in the 160–200 GHz band, with a minimum conversion loss of 8.62 dB near the center frequency and a relatively high loss at the band edges (maximum 11.94 dB), which overall meets the requirements of engineering applications. The performance fluctuation of the mixer is mainly caused by the impedance mismatch induced by fabrication tolerances. In addition, the parasitic parameters introduced by conductive adhesive and solder during assembly have an increasing influence with the rise in frequency, which further increases the link loss.

**Figure 17:**
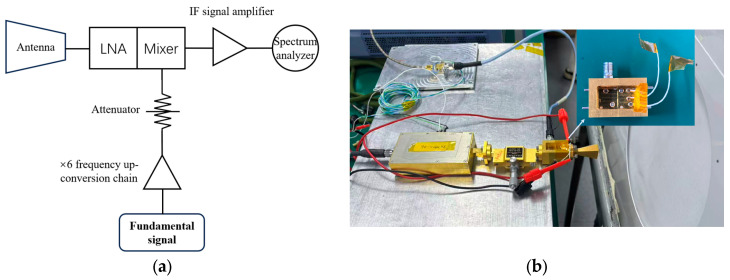
(**a**) Full-link noise test block diagram of the integrated system; (**b**) photo of the full-link noise test platform for the integrated system.

The schematic diagram of the full-link noise figure test platform for the integrated front-end is shown in Figure 17a. The full-link noise figure of the integrated frontend is measured by the Y-factor method [17]. The LO chain consists of a signal source followed by a sixth-frequency multiplier chain, with the output power adjusted to the appropriate drive level via an attenuator. After down-conversion by the mixer, the output IF signal is amplified by an IF amplifier, then received by a spectrum analyzer for parameter acquisition. The assembled noise figure test platform is shown in Figure 17b.

First, the antenna is aimed at free space (room temperature $T_{h}\approx 290\;K$), and the corresponding IF output power $P_{\mathrm{hot},\mathrm{dBm}}$ read by the spectrum analyzer is recorded. Subsequently, a cryogenic blackbody ($T_{c}=80\;K$) is placed in front of the antenna, and the corresponding IF output power $P_{\mathrm{cold},\mathrm{dBm}}$ read by the spectrum analyzer is recorded again. The relevant calculation expressions are given as follows:

Calculation Equation for the Y-factor:

(3) $$Y=10^{\frac{P_{\mathrm{hot},\mathrm{dBm}}-P_{\mathrm{cold},\mathrm{dBm}}}{10}}$$

Noise Temperature $T_{e}$ of the Receiver Frontend:

(4) $$T_{e}=\frac{T_{h}-YT_{c}}{Y-1}$$

Noise Figure NF:

(5) $$NF=10lg(1+\frac{T_{e}}{290K})$$

**Figure 18:**
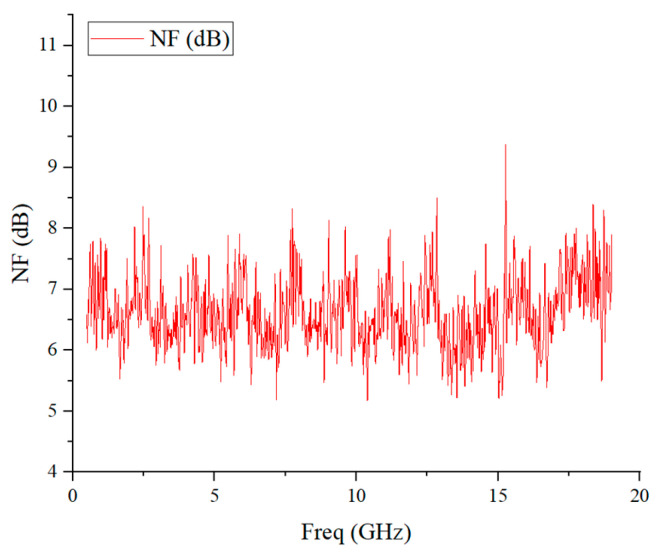
Noise figure test results.

After the test, the noise measurement results of the integrated frontend are sorted and calculated, as shown in Figure 18. The measurement results demonstrate that the detachable integrated frontend achieves an average full-link noise figure of 6.68 dB within the operating band, with stable cascaded performance, which verifies the cooperative working capability of the low-noise amplification and mixing modules.

The noise figure of the module is mainly attributed to the additional noise introduced by the peripheral circuits. The waveguide-to-microstrip transition and gold wire bonding introduce high-frequency parasitic loss and conductor loss; the quartz substrate and shielding cavity bring dielectric loss and ohmic loss; the DC bias network introduces a small amount of thermal noise. These additional losses, after being amplified, collectively elevate the overall noise figure of the module.

## 4. Conclusions

This paper presents a 183 GHz detachable terahertz radio frequency frontend, which integrates two core functional modules, namely a low-noise amplifier and a second-order subharmonic mixer, through a modular detachable integrated structure. A hybrid docking scheme combining positioning pins and screws is proposed to realize precise module alignment and rapid assembly/disassembly, effectively balancing high-frequency transmission performance and engineering maintainability.

Measurements show that within the 160–200 GHz band, the low-noise amplifier achieves an average gain of 16.51 dB, the second-order subharmonic mixer exhibits an average conversion loss of 10.37 dB, and the full-link noise figure of the integrated system is 6.68 dB.

The experimental results verify that the detachable integration scheme proposed in this study successfully realizes a terahertz radio frequency front-end with both high performance and favorable testability. In contrast to the shortcomings of conventional monolithic packaging architectures, including poor post-maintenance, irreversible assembly in monolithic integration, and bulky configuration in traditional discrete modular structures, the proposed scheme achieves repeated disassembly, assembly, and flexible replacement of modules via precision positioning and docking design, which effectively balances the high-frequency performance, assembly complexity, and engineering maintainability of the terahertz front-end. This scheme provides a feasible technical route for the design of high-performance and highly practical modular integration in the terahertz band. Future work can further expand the categories of integrated functional modules and continuously optimize the high-frequency performance of the system. The proposed scheme exhibits promising engineering application value in terahertz atmospheric radiometers, high-precision radars, and 6G communication receiver systems.

## Figures and Tables

**Table 1:** Comparative analysis of advantages and limitations of RF frontend integration architectures.

Application Frequency Band	Integration Scheme	Advantages	Limitations
Microwave and Millimeter-Wave	2.5D Packaging Integration Technology	Mature process, low cost	Low integration level, high high-frequency loss
Microwave and Millimeter-Wave	3D Integration Technology	High fabrication accuracy, high integration level, excellent passive performance	High process threshold, high cost, high high-frequency loss
Terahertz Wave	Monolithic Integration Technology	Short interconnection path, excellent high-frequency performance, high integration level	Permanently fixed packaging, difficult fault location, poor maintainability
Terahertz Wave	Detachable Integration Technology	Balances high-frequency high performance of terahertz and maintainability, convenient assembly and disassembly, supports independent testing, strong engineering adaptability	Slightly lower integration level than monolithic integrated packaging, requires strict control of assembly tolerance

## Data Availability

The data that support the findings of this study are available from the corresponding author upon reasonable request.
